# Authors’ reply: A norovirus intervariant GII.4 recombinant in Victoria, Australia, June 2016: the next epidemic variant? Reflections and a note of caution

**DOI:** 10.2807/1560-7917.ES.2016.21.41.30373

**Published:** 2016-10-13

**Authors:** Leesa Bruggink, Michael Catton, John Marshall

**Affiliations:** 1 Victorian Infectious Diseases Reference Laboratory, Royal Melbourne Hospital, The Peter Doherty Institute for Infection and Immunity, Melbourne, Victoria, Australia

**Keywords:** Norovirus, epidemic, new variant

**To the editor:** We thank the authors of the letter to the editor for their interest in our report of the detection of a potential new epidemic strain of norovirus [1]. The authors express some doubt as to the validity of our claims of a possible new norovirus GII.4 variant, since the recombinant form (GII.4_NewOrleans_2009/GII.4_Sydney_2012) has been reported previously in a number of instances, including by their own laboratory [2]. Nevertheless we stand by our proposal that a potential new epidemic strain of GII.4 norovirus has arisen, for the reasons outlined below.

Firstly, we proposed that the new epidemic variant is not the recombinant itself, but a derivative of it that has altered enough, we think, to evade herd immunity. The recombinant was not claimed to be in itself ‘new’, but only the precursor of the altered version with epidemic potential. The ORF1-ORF2 sequence submitted to GenBank, as cited in our publication [1], was of the *first detection of the recombinant* in Victoria, Australia, and was *not* the proposed new variant. That sequence was used to properly establish the existence of the recombinant, as the sequence bridges both ORF1 and ORF2 in one fragment. A full capsid sequence of the altered form of the recombinant was lodged in GenBank (KX767083) and, as cited in our publication [1], is 96.3% similar to its closest counterpart Sydney_2012, which is within the range of nucleotide difference presented by previous established epidemic variants, as calculated in our publication [1].

Regarding only referencing work at the Centers for Disease Prevention and Control (CDC) in the United States [3] for previous detection of the recombinant form, the reference was used purely because the data refer to an altered version of Sydney_2012, the ‘Sydney_2015’ strain. In fact, the data [3] do not actually refer to the recombinant (GII.4_NewOrleans_2009/GII.4_Sydney_2012), as the work only appears to be based on ORF2 data, as stated in our publication [1]. Other references to the recombinant form (GII.4_NewOrleans_2009/GII.4_Sydney_2012) were not cited, as the publication was primarily about the possible detection of an *altered form* of the recombinant, rather than the recombinant itself, and, importantly, highlighted the time delay between detection of a new variant and the resultant epidemic. We are not aware of any publication, other than the CDC data [3], that refers to an altered version of the recombinant or of GII.4_Sydney_2012 (ORF2) in general.

Finally, we agree that non-structural viral proteins may not have a direct role in a virus escaping herd immunity, but there is a sizeable literature on non-structural viral proteins playing important roles in viral virulence, of which the influenza non-structural protein 1 is but one well-characterised example [4].
